# A network meta-analysis of eight chemotherapy regimens for treatment of advanced ovarian cancer

**DOI:** 10.18632/oncotarget.13253

**Published:** 2016-11-09

**Authors:** Xi-Ping Jiang, Xiao-Hui Rui, Cai-Xia Guo, Ya-Qing Huang, Qin Li, Yun Xu

**Affiliations:** ^1^ Department of Gynecology, the First People's Hospital of Changzhou, Changzhou 213003, P. R. China

**Keywords:** advanced ovarian cancer, chemotherapy, combination therapy, network meta-analysis, bayesian network model

## Abstract

This study compared the short-term efficacies of different chemotherapy regimens in the treatment of advanced ovarian cancer (AOC) through pair-wise and network meta-analyses (NMA). Randomized controlled trials (RCTs) identified in a comprehensive online literature search met our inclusion criteria. Direct and indirect evidence was combined to compare odds ratios (OR) and surfaces under the cumulative ranking curves (SUCRA) across the different treatment regimens. Twelve eligible RCTs were finally included, involving eight regimens (Paclitaxel + Carboplatin [PC], Gemcitabine + Carboplatin [GC], Carboplatin, Pegylated Liposomal Doxorubicin + Carboplatin [PLD + Carboplatin], Paclitaxel, Paclitaxel + Carboplatin + Topotecan [PC + Topotecan], Paclitaxel + Carboplatin + Epirubicin [PC + Epirubicin] and Docetaxel + Carboplatin [DC]). The NMA results revealed that in terms of overall response rate (ORR) and disease control rate (DCR), PC (ORR: OR=2.59, 95%CI=1.20–6.22; DCR: OR=2.58, 95%CI=1.05–6.82) and GC (ORR: OR=2.08, 95%CI=1.08–4.37; DCR: OR=2.43, 95%CI=1.07–5.80) were more effective against AOC than Carboplatin alone. Similarly, PC (OR=0.21, 95%CI=0.05–0.69), GC (OR=0.31, 95%CI=0.09–0.90) and PLD + Carboplatin (OR=0.22, 95%CI=0.04–0.92) slowed disease progression better than Carboplatin alone. We also found that PC was more efficacious against AOC than Carboplatin or Paclitaxel single-agent chemotherapy. Combination chemotherapy is thus recommended for AOC, and should guide subsequent drug development and treatment strategies.

## INTRODUCTION

Ovarian cancer (OC) is the second most common gynecological cancer in women, after endometrial cancer [1]. Some 238,719 OC cases were reported in 2012, with 151,917 deaths worldwide [2]. Although the etiology of OC is not yet fully understood, certain factors, including age, late childbearing, early onset at menarche, late menopause, and *breast cancer 1* (*BRCA1*) and *breast cancer 2* (*BRCA2*) mutations, are implicated in OC formation and development [3, 4]. Environmental factors also play roles in OC promotion, and include dietary habits, air pollution, alcohol consumption, and pathogen infection [5]. In addition, lack of early diagnosis may be a key factor in disease progression. According to a previous study, only 25% of patients were diagnosed with early-stage OC while 58% and 17% of cases were diagnosed with stage III and IV disease, with 10-year survival rates of 21% and <5%, respectively [6]. Thus, early diagnosis and timely treatment are of crucial importance in overcoming this disease.

Chemotherapy is still a commonly used approach in OC treatment. With respect to mechanism of action, such chemotherapeutics may selectively modify and inhibit their targets. For instance, Epirubicin inhibits cell division through inhibition of DNA and RNA synthesis [7], and Docetaxel aids T cell in recognizing tumor cells via modification of the tumor phenotype [8]. Alternatively, chemotherapy drugs may exert toxic effects on tumor cells, inducing cell damage or disruption of tumor cell differentiation; such drugs include Paclitaxel [9] and Pegylated Liposomal Doxorubicin (PLD) [10]. Other OC chemotherapeutics, such as Gemcitabine [11] and Topotecan [12], inhibit specific signaling pathways and certain cell functions in tumor cells. Combination therapy has been widely recommended for OC patient treatment. Cisplatin or Carboplatin is usually combined with alkyl compounds like Cyclophosphamide. While Cisplatin and Carboplatin are equally efficacious, Carboplatin is less toxic than Cisplatin. Thus, Carboplatin is commonly used in combination with Paclitaxel [13] or PLD [14]. Nevertheless, few studies have compared and evaluated the efficacy of different first-line regimens in treating OC. With the introduction of these therapeutic options, and the lack of randomized trials that directly compare all available chemotherapy regimens, it was of interest to indirectly compare the relative efficacy and safety of these chemotherapy regimens using a network meta-analysis [15–16].

Network meta-analysis is also known as multiple-treatments comparison and can synthesize data from both direct (within-trial comparisons) as well as indirect comparisons (inter-trial treatment comparisons using a common comparator treatment) of diverse regimens [17]. Furthermore, the Bayesian approach can estimate the rank probability that, each of the regimens is the best, the second best, and so on [18]. It is highly advocate that investigators should consider all potentially relevant data when comparing treatments and multiple-treatment comparisons is consistent with the true situation when using a wide network of studies that are included appropriately [19]. This study included RCTs published up to December 2015 involving eight chemotherapy regimens for advanced ovarian cancer (AOC) treatment including Carboplatin, Paclitaxel, Paclitaxel + Carboplatin (PC), Gemcitabine + Carboplatin (GC), PLD + Carboplatin, PC + Topotecan, PC + Epirubicin and Docetaxel + Carboplatin (DC). It is believed that this network meta-analysis can provide some useful information about comparison between these first-line regimens agents for AOC through integrating and indirect methods, expecting this message will be helpful for physicians and patients in decision-making.

## RESULTS

### Baseline characteristics of included studies

The reviewers initially identified 2,664 records from database searches, of which 2,578 and 86 were collected via key word searches and manual retrieval, respectively. We excluded 51 duplicate studies, 633 letters or reviews, 175 non-human studies, and 155 non-English language publications. From the remaining 1,650 studies, we further excluded 678 non-cohort studies, 583 irrelevant to AOC, 373 unrelated to chemotherapy, and 4 for no available data or missing data. Finally, 12 RCTs met our inclusion criteria and were deemed eligible for meta-analysis [20–31] (Supplementary Figure 1). The study included 6,187 patients with AOC, the majority of whom received the PC chemotherapy regimen. The included RCTs were published between 2004 and 2015, and all were two-arm trials. Eleven out of 12 assessed Caucasians and one assessed Asians. RCT baseline characteristics are provided in Supplementary Table 1 and bias assessment by the Cochrane Collaboration's tool is shown in Supplementary Figure 2.

### Pairwise meta-analysis

The short-term efficacy of eight AOC chemotherapy regimens was assessed via direct paired comparisons as follows: (1) ORR and DCR: PC was more effective than PC + Topotecan (OR=1.43, 95%CI=1.12–1.83; OR=1.40, 95%CI=1.08–1.81, respectively), and GC had better outcomes than Carboplatin single-agent chemotherapy (OR=2.00, 95%CI=1.30–3.08; OR=2.25, 95%CI=1.51–4.30, respectively); (2) ORR: PC was more effective than Carboplatin single-agent chemotherapy (OR=3.10, 95%CI=1.21–7.79); (3) PD: combination chemotherapy, such as PC or GC, slowed disease progression more effectively than Carboplatin single-agent chemotherapy (OR=0.11, 95%CI=0.02–0.51; OR=0.44, 95%CI=0.22–0.86, respectively) (Table 1); (4) CR: GC resulted in better outcomes than Carboplatin single-agent chemotherapy (OR=2.60, 95%CI=1.24–5.43); (5) SD: PC was more effective than PC + Epirubicin (OR=1.94, 95%CI=1.03–3.67). In terms of PR, all eight AOC treatment regimens performed the same (Supplementary Table 2).

**Table 1:** Estimated OR and 95%CI from pairwise meta-analysis of efficacy events in advanced ovarian cancer patients in terms of ORR, PD and DCR

Included studies	Comparisons	Efficacy events		Pairwise meta-analysis		
		Treatment1	Treatment2	OR (95%CI)	*I*^2^	*P_h_*
ORR						
Gordon AN(2011)[23]	A vs. B	81/114	97/139	1.18 (0.69-2.01)	NA	NA
Gonzalez-Martin AJ(2005)[30]	A vs. C	31/41	20/40	**3.10 (1.21-7.79)**	NA	NA
Mahner S(2015)[20] Gladieff L(2012)[22] Bafaloukos D(2010)[25]	A vs. D A vs. D A vs. D	187/407	165/385	1.14 (0.86-1.51)	0.0%	0.403
Lortholary A(2012)[21]	A vs. E	19/51	20/57	1.10 (0.50-2.41)	NA	NA
Pfisterer J(2006)[27]	A vs. F	495/650	454/658	**1.43 (1.12-1.83)**	NA	NA
du Bois A(2006)[28]	A vs. G	381/635	389/647	0.99 (0.80-1.24)	NA	NA
Mori T(2007)[26] Vasey PA(2004)[31]	A vs. HA vs. H	180/312	182/313	0.99 (0.72-1.36)	0.0%	0.687
Pfisterer J(2005)[29]	B vs. C	84/178	55/178	**2.00 (1.30-3.08)**	NA	NA
**PD**						
Gordon AN(2011)[23]	A vs. B	11/114	14/139	0.95 (0.42-2.19)	NA	NA
Gonzalez-Martin AJ(2005)[30]	A vs. C	2/41	13/40	**0.11 (0.02-0.51)**	NA	NA
Mahner S(2015)[20] Gladieff L(2012)[22] Bafaloukos D(2010)[25]	A vs. D A vs. D A vs. D	32/407	31/385	0.95 (0.56-1.59)	0.0%	0.969
Lortholary A(2012)[21]	A vs. E	13/51	15/57	0.96 (0.40-2.27)	NA	NA
Bolis G(2010)[24] Pfisterer J(2006)[27]	A vs. F A vs. F	28/820	31/814	0.88 (0.52-1.48)	0%	0.910
du Bois A(2006)[28]	A vs. G	19/635	21/647	0.92 (0.49-1.73)	NA	NA
Mori T(2007)[26] Vasey PA(2004)[31]	A vs. H A vs. H	31/312	29/313	1.08 (0.64-1.85)	0.0%	0.389
Pfisterer J(2005)[29]	B vs. C	14/178	29/178	**0.44 (0.22-0.86)**	NA	NA
**DCR**						
Gordon AN(2011)[23]	A vs. B	97/114	116/139	1.13 (0.57-2.24)	NA	NA
Gonzalez-Martin AJ(2005)[30]	A vs. C	33/41	25/40	2.48 (0.91-6.75)	NA	NA
Gladieff L(2012)[22] Bafaloukos D(2010)[25]	A vs. D A vs. D	215/279	189/254	1.14 (0.77-1.71)	0.0%	0.383
Lortholary A(2012)[21]	A vs. E	34/51	33/57	1.45 (0.66-3.19)	NA	NA
Pfisterer J(2006)[27]	A vs. F	513/650	479/658	**1.40 (1.08-1.81)**	NA	NA
du Bois A(2006)[28]	A vs. G	410/635	404/647	1.10 (0.87-1.38)	NA	NA
Mori T(2007)[26]	A vs. H	7/16	6/13	0.91 (0.21-3.95)	NA	NA
Pfisterer J(2005)[29]	B vs. C	152/178	124/178	**2.25 (1.51-4.30)**	NA	NA

Notes: ORR=overall response rate; PD=progressive disease; DCR= disease control rate; OR=odd ratios; 95%CI=95% confidence intervals; NA=not available; T=treatment; A= Paclitaxel+Carboplatin; B= Gemcitabine+Carboplatin; C= Carboplatin; D= Pegylated liposomal doxorubicin+Carboplatin; E= Paclitaxel; F= Paclitaxel+Carboplatin+Topotecan; G= Paclitaxel+ Carboplatin +Epirubicin; H= Docetaxel+Carboplatin.

### Network evidence

**Figure 1:**
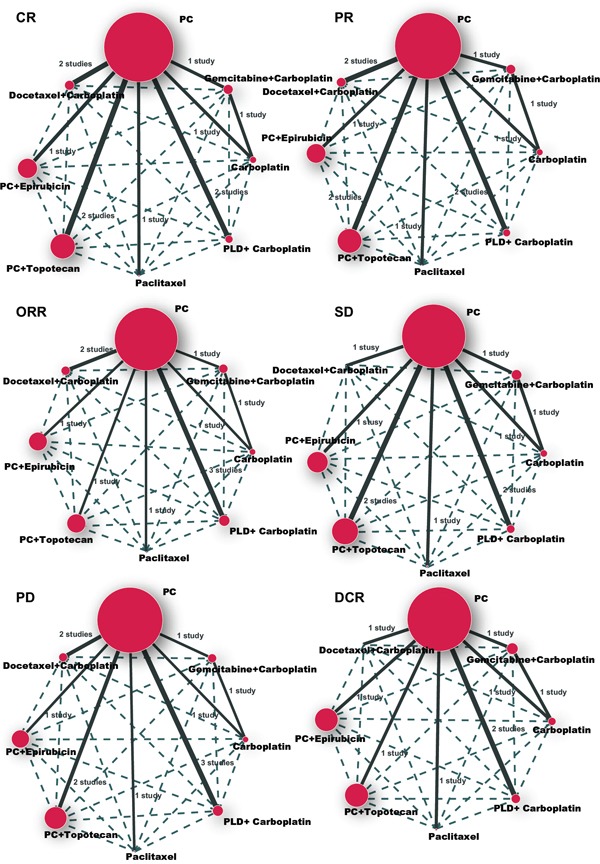
CR, PR, ORR, SD, PD and DCR network plot CR = complete response; PR = partial response; ORR = overall response rate; SD = stable disease; PD = progressive disease; DCR = disease control rate; A = Paclitaxel + Carboplatin; B = Gemcitabine + Carboplatin; C = Carboplatin; D = Pegylated liposomal doxorubicin + Carboplatin; E = Paclitaxel; F = Paclitaxel + Carboplatin + Topotecan; G = Paclitaxel + Carboplatin + Epirubicin; H = Docetaxel + Carboplatin.

The majority of patients received the PC regimen. With respect to CR, PR, ORR, PD, SD, and DCR assessment, there were more direct paired comparisons performed for the PC and PLD + Carboplatin regimens than for the other regimens (Figure 1).

### Inconsistency tests

Inconsistency tests were performed via the node-splitting method for the six endpoint outcomes (CR, PR, ORR, PD, SD, and DCR). Direct and indirect evidences showed consistency for all endpoint outcomes, and so the consistency model was adopted (both *P*>0.05) (Table 2).

**Table 2:** OR values and *P* values of direct and indirect pairwise comparisons of eight treatment modalities under six endpoint outcomes

Pairwise comparisons	Direct OR values						Indirect OR values						*P* values					
	CR	PR	ORR	SD	PD	DCR	CR	PR	ORR	SD	PD	DCR	CR	PR	ORR	SD	PD	DCR
B vs. A	0.89	0.96	0.85	1.20	1.10	0.88	1.80	0.66	0.63	3.10	4.60	0.98	0.501	0.623	0.708	0.430	0.239	0.905
C vs. A	0.68	0.43	0.31	3.20	2.50	0.39	0.33	0.67	0.43	1.20	2.50	0.35	0.427	0.590	0.664	0.446	0.200	0.884
C vs. B	0.38	0.67	0.49	1.00	2.30	0.39	0.73	0.46	0.36	2.90	3.00	0.45	0.505	0.633	0.668	0.456	0.206	0.919

Notes: CR=complete response; PR=partial response; ORR=overall response rate; SD=stable disease; PD=progressive disease; DCR=disease control rate; OR=odd ratios; A= Paclitaxel+Carboplatin; B= Gemcitabine+Carboplatin; C= Carboplatin.

### Network meta-analyses

**Figure 2:**
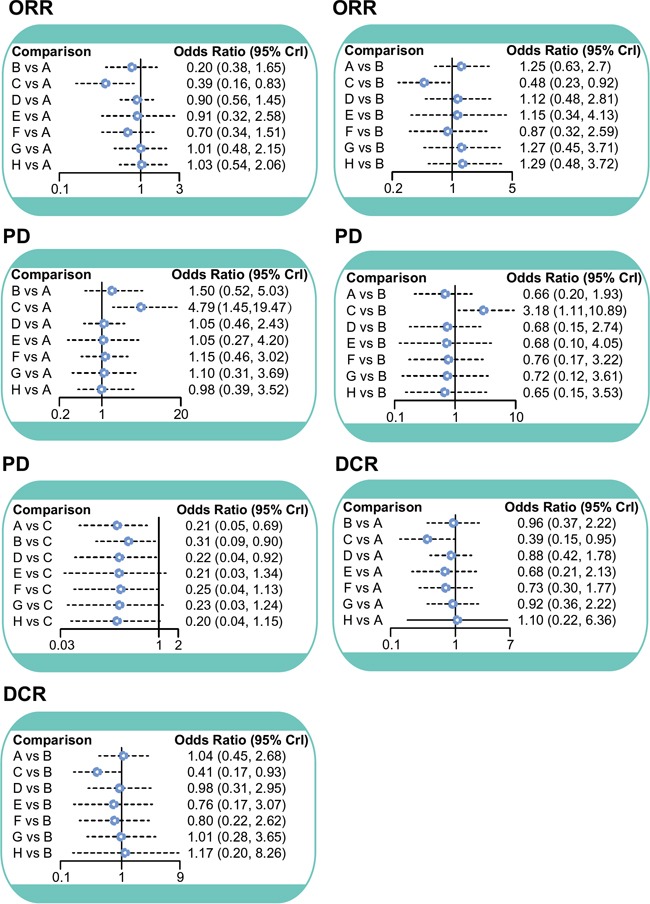
ORR, PD and DCR forest plot ORR = overall response rate; PD = progressive disease; DCR = disease control rate; A = Paclitaxel + Carboplatin; B = Gemcitabine + Carboplatin; C = Carboplatin; D = Pegylated liposomal doxorubicin + Carboplatin; E = Paclitaxel; F = Paclitaxel + Carboplatin + Topotecan; G = Paclitaxel + Carboplatin + Epirubicin; H = Docetaxel + Carboplatin.

The NMA revealed that PC showed higher efficacy in the treatment of AOC than Carboplatin (ORR [overall response rate]: OR=2.59, 95%CI =1.20~6.22; DCR [disease control rate]: OR=2.58, 95%CI=1.05~6.82). As also compared to the Carboplatin, GC exhibited higher efficacy in the treatment of AOC (ORR: OR=2.08, 95%CI =1.08~4.37; DCR: OR=2.43, 95%CI=1.07~5.80). With respect to PD, PC, GC, and PLD + Carboplatin slowed disease progression more effectively than Carboplatin single-agent chemotherapy (OR=0.21, 95%CI=0.05–0.69; OR=0.31, 95%CI=0.09–0.90; OR=0.22, 95%CI=0.04–0.92, respectively) (Table 3, Figure 2). With respect to PR, CR, and SD, all eight chemotherapy regimens performed the same (Supplementary Table 3).

**Table 3:** Odds ratios and 95% confidence intervals of eight drugs in the treatment of advanced ovarian cancer in terms of ORR, PD and DCR

Odds ratios (95% confidence intervals)							
**ORR**							
**PC**	0.80 (0.38, 1.65)	**0.39 (0.16, 0.83)**	0.90 (0.56, 1.45)	0.91 (0.32, 2.58)	0.70 (0.34, 1.51)	1.01 (0.48, 2.15)	1.03 (0.54, 2.06)
1.25 (0.61, 2.65)	**GC**	**0.48 (0.23, 0.92)**	1.12 (0.48, 2.81)	1.15 (0.34, 4.13)	0.87 (0.32, 2.59)	1.27 (0.45, 3.71)	1.29 (0.48, 3.72)
**2.59 (1.20, 6.22)**	**2.08 (1.08, 4.37)**	**Carboplatin**	2.33 (0.97, 6.59)	2.37 (0.67, 9.44)	1.81 (0.65, 5.75)	3.01(0.71-13.10)	2.69 (1.00, 8.38)
1.11 (0.69, 1.77)	0.89 (0.36, 2.08)	0.43 (0.15, 1.03)	**PLD+ Carboplatin**	1.02 (0.33, 3.09)	0.78 (0.32, 1.91)	1.12 (0.46, 2.65)	1.15 (0.50, 2.71)
1.10 (0.39, 3.12)	0.87 (0.24, 2.94)	0.42 (0.11, 1.48)	0.99 (0.32, 3.07)	**Paclitaxel**	0.77 (0.21, 2.79)	1.14 (0.31, 3.85)	1.14 (0.34, 3.93)
1.43 (0.66, 2.94)	1.15 (0.39, 3.17)	0.55 (0.17, 1.55)	1.28 (0.52, 3.10)	1.30 (0.36, 4.67)	**PC+Topotecan**	1.46 (0.48, 4.19)	1.46 (0.54, 4.22)
0.99 (0.46, 2.06)	0.79 (0.27, 2.23)	0.38 (0.12, 1.07)	0.89 (0.38, 2.16)	0.88 (0.26, 3.23)	0.69 (0.24, 2.09)	**PC+Epirubicin**	1.02 (0.38, 2.83)
0.97 (0.48, 1.86)	0.78 (0.27, 2.07)	0.37 (0.12, 1.00)	0.87 (0.37, 2.00)	0.88 (0.25, 2.98)	0.68 (0.24, 1.86)	0.98 (0.35, 2.64)	**DC**
**PD**							
**PC**	1.50 (0.52, 5.03)	**4.79 (1.45, 19.47)**	1.05 (0.46, 2.43)	1.05 (0.27, 4.20)	1.15 (0.46, 3.02)	1.10 (0.31, 3.69)	0.98 (0.39, 3.52)
0.66 (0.20, 1.93)	**GC**	**3.18 (1.11, 10.89)**	0.68 (0.15, 2.74)	0.68 (0.10, 4.05)	0.76 (0.17, 3.22)	0.72 (0.12, 3.61)	0.65 (0.15, 3.53)
**0.21 (0.05, 0.69)**	**0.31 (0.09, 0.90)**	**Carboplatin**	**0.22 (0.04, 0.92)**	0.21 (0.03, 1.34)	0.25 (0.04, 1.13)	0.23 (0.03, 1.24)	0.20 (0.04, 1.15)
0.96 (0.41, 2.15)	1.47 (0.36, 6.50)	**4.57 (1.09, 23.70)**	**PLD+ Carboplatin**	0.98 (0.19, 4.77)	1.10 (0.30, 3.82)	1.05 (0.24, 4.27)	0.95 (0.27, 4.44)
0.95 (0.24, 3.73)	1.47 (0.25, 9.71)	4.65 (0.75, 39.99)	1.02 (0.21, 5.20)	**Paclitaxel**	1.12 (0.20, 5.61)	1.05 (0.15, 6.55)	0.95 (0.19, 6.50)
0.87 (0.33, 2.17)	1.32 (0.31, 6.01)	4.05 (0.89, 22.62)	0.91 (0.26, 3.32)	0.89 (0.18, 4.92)	**PC+Topotecan**	0.93 (0.19, 4.32)	0.84 (0.23, 4.28)
0.91 (0.27, 3.26)	1.39 (0.28, 8.15)	4.36 (0.81, 32.25)	0.95 (0.23, 4.24)	0.95 (0.15, 6.54)	1.08 (0.23, 5.15)	**PC+Epirubicin**	0.90 (0.21, 5.63)
1.02 (0.28, 2.54)	1.55 (0.28, 6.74)	4.94 (0.87, 24.98)	1.05 (0.22, 3.72)	1.05 (0.15, 5.39)	1.19 (0.23, 4.31)	1.11 (0.18, 4.68)	**DC**
**DCR**							
**PC**	0.96 (0.37, 2.22)	**0.39 (0.15, 0.95)**	0.88 (0.42, 1.78)	0.68 (0.21, 2.13)	0.73 (0.30, 1.77)	0.92 (0.36, 2.22)	1.10 (0.22, 6.36)
1.04 (0.45, 2.68)	**GC**	**0.41 (0.17, 0.93)**	0.98 (0.31, 2.95)	0.76 (0.17, 3.07)	0.80 (0.22, 2.62)	1.01 (0.28, 3.65)	1.17 (0.20, 8.26)
**2.58 (1.05, 6.82)**	**2.43 (1.07, 5.80)**	**Carboplatin**	2.40 (0.72, 7.51)	1.82 (0.39, 7.78)	1.88 (0.53, 6.91)	2.34 (0.68, 8.91)	2.92 (0.43, 20.09)
1.13 (0.56, 2.38)	1.03 (0.34, 3.25)	0.42 (0.13, 1.38)	**PLD+ Carboplatin**	0.75 (0.21, 3.05)	0.78 (0.32, 1.91)	1.01 (0.33, 3.32)	1.26 (0.23, 7.76)
1.47 (0.47, 4.72)	1.32 (0.33, 5.93)	0.55 (0.13, 2.56)	1.34 (0.33, 4.71)	**Paclitaxel**	1.04 (0.26, 4.66)	1.32 (0.31, 5.63)	1.65 (0.23, 12.58)
1.37 (0.57, 3.35)	1.26 (0.38, 4.46)	0.53 (0.14, 1.88)	1.25 (0.37, 3.89)	0.96 (0.21, 3.91)	**PC+Topotecan**	1.25 (0.35, 4.62)	1.52 (0.26, 10.48)
1.08 (0.45, 2.78)	0.99 (0.27, 3.59)	0.43 (0.11, 1.48)	0.99 (0.30, 3.01)	0.76 (0.18, 3.22)	0.80 (0.22, 2.86)	**PC+Epirubicin**	1.23 (0.20, 8.53)
0.91 (0.16, 4.45)	0.86 (0.12, 5.09)	0.37 (0.12, 1.00)	0.79 (0.13, 4.39)	0.61 (0.08, 4.36)	0.66 (0.10, 3.92)	0.81 (0.12, 5.09)	**DC**

Notes: Odds ratios and 95% confidence intervals below the treatments should be read from row to column while above the treatments should be read from column to row. OR > 1 favors the line-defining treatment, in ORR and DCR section, OR > 1 favors the row-defining treatment, while in PD section, OR > 1 favors the column-defining treatment. Results with evidence of benefit are in bold and underlined. ORR=overall response rate; PD=progressive disease; DCR= disease control rate; PC= Paclitaxel+Carboplatin; GC= Gemcitabine+Carboplatin; PLD= Pegylated liposomal doxorubicin; DC= Docetaxel+Carboplatin.

### Surface under the cumulative ranking curves (SUCRA)

With respect to the six endpoint outcomes, the efficacies of all eight AOC chemotherapy regimens were determined via SUCRA values, with the following results: (1) PC had the highest SUCRA values in terms of CR (74.0%), PD (69.6%) and DCR (74.0%); (2) Paclitaxel showed the highest SUCRA value with respect to PR (73.4%); (3) DC (73.1%) ranked highest in terms of ORR, followed by PC (73.0%); (4) PC + Epirubicin had the highest SUCRA value for SD (80.8%); (5) Carboplatin single-agent chemotherapy had the lowest SUCRA values for all six endpoint outcomes (CR: 19.5%; PR: 22.9%; ORR: 15.6%; SD: 31.0%; PD: 14.8; DCR: 19.5%) (Table 4).

**Table 4:** SUCRA values of eight treatment modalities under six endpoint outcomes

Treatments	SUCRA values					
	CR	PR	ORR	SD	PD	DCR
**A**	**0.740**	0.644	0.730	0.538	**0.696**	**0.740**
**B**	0.660	0.531	0.520	0.378	0.463	0.660
**C**	0.195	0.229	0.156	0.310	0.148	0.195
**D**	0.639	0.445	0.605	0.446	0.648	0.638
**E**	0.453	**0.734**	0.610	0.674	0.641	0.453
**F**	0.458	0.600	0.396	0.644	0.593	0.458
**G**	0.663	0.679	0.716	**0.808**	0.613	0.663
**H**	0.698	0.650	**0.731**	0.704	0.694	0.698

Notes: CR=complete response; PR=partial response; ORR=overall response rate; SD=stable disease; PD=progressive disease; DCR=disease control rate; SUCRA= surface under the cumulative ranking curves; A= Paclitaxel+Carboplatin; B= Gemcitabine+Carboplatin; C= Carboplatin; D= Pegylated liposomal doxorubicin+Carboplatin; E= Paclitaxel; F= Paclitaxel+Carboplatin+Topotecan; G= Paclitaxel+ Carboplatin +Epirubicin; H= Docetaxel+Carboplatin.

### Cluster analyses

**Figure 3:**
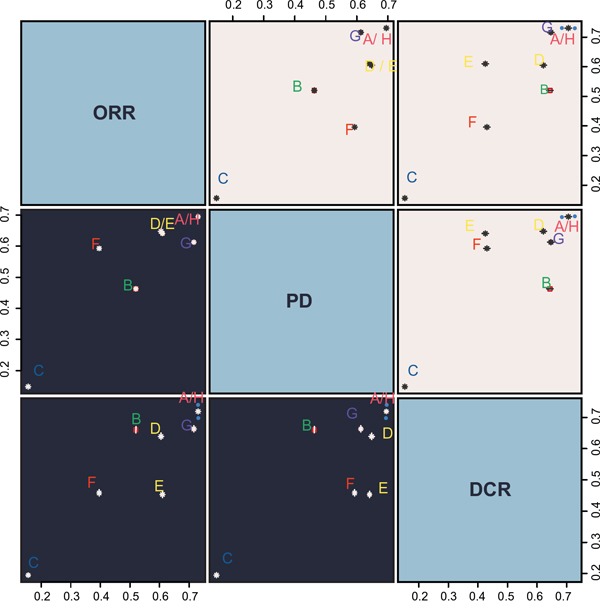
ORR, PD and DCR cluster analysis diagram ORR = overall response rate; PD = progressive disease; DCR = disease control rate; A = Paclitaxel + Carboplatin; B = Gemcitabine + Carboplatin; C = Carboplatin; D = Pegylated liposomal doxorubicin + Carboplatin; E = Paclitaxel; F = Paclitaxel + Carboplatin + Topotecan; G = Paclitaxel + Carboplatin + Epirubicin; H = Docetaxel + Carboplatin.

Cluster analyses of ORR, PD and DCR SUCRA values showed that PC had the highest efficacy against AOC, followed by DC, while Carboplatin single-agent chemotherapy had the lowest efficacy (Figure 3).

## DISCUSSION

In this study, direct pairwise meta-analyses and NMA were conducted to compare and evaluate the efficacies of eight widely used chemotherapy regimens (Carboplatin single-agent chemotherapy, Paclitaxel single-agent chemotherapy, PC, GC, PLD + Carboplatin, PC + Topotecan, PC + Epirubicin and DC) in the treatment of AOC. Direct pairwise meta-analysis and NMA results showed that the PC regimen was more effective in treating AOC than the other regimens. PC is a relatively common first-line chemotherapy regimen. Carboplatin, a commonly used adjuvant with acceptable toxicities, has shown great efficacy in combination therapy [32]. Pacilitaxel promotes stable microtubule assembly by acting specifically at the beta-tubulin subunit N-terminus [33], impeding depolymerization and inhibiting cancer cell division [9]. In terms of progression-free survival, previous studies showed that PC was more efficacious than GC [23], while no difference was detected when PC was compared to PLD + Carboplatin [20, 34], PC + Topotecan [35], PC + Epirubicin [36, 37] or DC [26]. According to our NMA SUCRA values, PC produced better outcomes with regard to CR, PD and DCR, while DC was more effective regarding ORR and PC + Epirubicin had a higher efficacy in terms of DCR. These results are consistent with previous studies [26, 37, 38], demonstrating there was no efficacy difference between PC, PC + Epirubicin, and DC. However, some studies indicated that toxicity was lower in PC as compared to PC + Epirubicin [37], PC + Topotecan [38], and DC [26]. Epirubicin inhibits DNA and RNA synthesis by intercalating DNA strands [7] and Topotecan inhibits cancer cell differentiation through PPARγ degradation [12], which disturbs the normal processes of cell division and differentiation, and impedes cell damage repair,. The mechanisms underlying high DC toxicity have not yet been reported. In sum, PC exhibited reduced toxicity and fewer side effects as compared to the other studied regimens.

Both pairwise meta-analysis and NMA indicated that Carboplatin single-agent chemotherapy was less effective than the other regimens. Carboplatin, a Cisplatin analogue, is widely used as an adjuvant drug for cancer treatment due to its lower toxicity and reduced side effects [39]. Carboplatin single-agent chemotherapy was reportedly highly effective and well tolerated in patients with early stage cancer, and was suitable for long-term use [40]. However, for patients with advanced-stage cancer, Carboplatin alone might be less effective than other regimens [20]. Previous studies showed that combination chemotherapy, such as PC or GC, was more effective than Carboplatin alone [30, 41]. Therefore, Carboplatin should be combined with other drugs to treat advanced cancer patients.

In addition, as the toxicity of the eight chemotherapy regimens was mentioned above, the adverse events also are an object of concern. There were very little or no patients received PC in the moderate, severe or life threating degrees of adverse events including allergy, anorexia, arthralgia, fatigue, febrile neutropenia, nausea, neurotoxicity, mucositis, vomiting, thrombosis and haemoglobin [24]. And patients treated by GC had more frequent hematologic toxicities of grade three or four than carboplatin, in which neutropenia was the predominant toxicity [27]. Whilst, the early discontinuation leaded by severe nonhematologic toxicity, grade two or greater alopecia, hypersensitivity reactions and sensory neuropathy occurred more frequently in the patients received Paclitaxel and Carboplatin than PLD + Carboplatin [34]. Meanwhile, the risk of drug-related serious adverse events was higher in the patients received PC + Topotecan than those received PC [24]. And PC + Epirubicin for AOC had more frequently occurrences of grade three or four hematologic and some nonhematologic toxicity (nausea/emesis, mucositis, and infections). than DC [28]. However, the data of the adverse events of the eight chemotherapy regimens was not very complete, so the network comparison couldn't be performed.

This meta-analysis was limited by several factors: (1) the small number of included references restricts the generality of the research results to some extent; (2) cluster analysis results were not significant enough to fully support the research summaries; (3) the data of the safety outcomes was so incomplete in enrolled studies that this study couldn't give a statistical analysis to compare the safety of eight chemotherapy regimens on AOC. In support of the study's conclusions: (1) this study included a sufficiently large number of individual patient cases; (2) various comparisons showed consistency; (3) our meta-analysis results were consistent with those of previous studies.

In conclusion, this study demonstrated that PC was more effective against AOC than any of the other studied regimens. Carboplatin single-agent chemotherapy was least effective. Thus, combination chemotherapy is recommended for treatment of AOC, and this should guide subsequent drug development and treatment strategies. Also, our study highlights the value of network meta-analysis for the treatment of AOC, providing indirect comparisons of multiple chemotherapy regimens for more valuable, comprehensive and complete results. Compared with a traditional meta-analysis, a network meta-analysis enables indirect comparison using a common comparator when a head-to-head trial is not accessible and also combines direct and indirect comparisons simultaneously for comparing several interventions.

## MATERIALS AND METHODS

### Literature search

A comprehensive literature search was performed online in Cochrane Library (from 1996 to December 2015) and PubMed (from April 2000 to December 2015). The search strategy was based on keywords and free words including ovarian cancer, pharmacotherapy, chemotherapy, Paclitaxel, Carboplatin and Gemcitabine, etc., in the combination with the Boolean logic AND, OR and NOT. Specific search strategy as follows: (((“Ovarian Neoplasms”[mh] OR Ovarian Malignant Tumor[tiab] OR Ovarian Cancer[tiab] OR Ovary Cancer[tiab] OR Cancer, Ovarian[tiab] OR Cancer of Ovary[tiab])) AND (“Drug Therapy”[mh] OR Medication Errors[tiab] OR Drug Administration Routes[tiab] OR Opiate Substitution Treatment[tiab] OR Polypharmacy [tiab] OR Medicine[tiab] OR Antineoplastic Drugs[tiab] OR Cancer Chemotherapy Agents[tiab] OR Anticancer Agents[tiab] OR Antitumor Agents[tiab] OR Chemotherapeutic Anticancer Drug[tiab])) AND (“randomized controlled trial”[pt] OR “controlled clinical trial”[pt] OR “randomized controlled trials as topic”[Mesh] OR “clinical trials as topic”[mh] OR “controlled clinical trials as topic”[mh] OR placebos[mh] OR “random allocation”[mh] OR “double-blind method”[mh] OR randomized[tiab] OR placebo[tiab] OR randomization[tiab] OR randomly allocated[tiab] OR ((double[tw] OR treble[tw] OR triple[tw]) AND (mask* [tw] OR blind* [tw]))). A manual search was also conducted to identify additional potentially relevant references.

### Study selection

Inclusion criteria included: (1) study design: randomized controlled trial (RCT); (2) interventions: Carboplatin single-agent chemotherapy, Paclitaxel single-agent chemotherapy, PC, GC, PLD + Carboplatin single-agent chemotherapy, PC + Topotecan, PC + Epirubicin, or DC; (3) study subject: AOC patients aged 19 – 89 years; (4) endpoints: complete response (CR), partial response (PR), overall response rate (ORR), progressive disease (PD), stable disease (SD), and disease control rate (DCR). Exclusion criteria included: (1) studies with insufficient data, such as non-paired studies; (2) non-RCTs; (3) duplicated publications; (4) meeting reports, systematic reviews or abstracts; (5) references irrelevant to AOC; (6) non-English publications; (7) non-human studies; (8) non-drug regimens.

### Data extraction and quality assessment

RCT data were extracted by independent reviewers using a form designed for this study. Four researchers conducted data extraction; Xi-Ping Jiang and Xiao-Hui Rui were responsible for the extraction of baseline data, while the depth data was extracted by Cai-Xia Guo and Yun Xu. Any disagreements were resolved by discussion with Xi-Ping Jiang, Xiao-Hui Rui, Cai-Xia Guo and Yun Xu. The Cochrane Collaboration's tool was used by more than two of our study authors to assess the risk of bias in each included RCT, including potential sources of bias included random allocation, allocation concealment, blinding, incomplete outcome data, selective outcome reporting, and other biases. Each potential bias source was assigned a judgment of “yes”, “no”, or “unclear” for each RCT. Then, the number of “unclear” or “no” judgments was calculated, and each RCT was classified as having a low, high, or unclear risk of bias as follows: 0–1, low risk; 2–3, moderate risk; ≥4, high risk [42]. Review Manager 5 (RevMan 5.2.3, Cochrane Collaboration, Oxford, UK) was employed for quality assessment and investigation of publication bias.

### Statistical analysis

First, direct comparisons across different treatment arms were performed using a traditional pairwise meta-analysis. Odd ratios (ORs) and 95% confidence intervals (CIs) were used to pool the estimates of intervention effects. Heterogeneity across different studies was examined using Chi-square and I-square tests [43]. Second, results were presented as a network plot in R version 3.2.1, with each node representing an intervention. Node sizes were associated with sample sizes, and the thickness of the line connecting any two nodes indicated the number of included studies. Third, comparisons of different treatments were executed using Bayesian NMA. According to non-informative priors, effect sizes and precision were specified in each analysis. Convergence and lack of auto-correlation were explored and verified after four chains and a 20,000-simulation burn-in phase, and direct probabilities were determined in an additional 50,000-simulation phase [44]. The node-splitting method was used for selection of a consistency or inconsistency model, via evaluation of the consistency between direct and indirect evidence [45]. For the interpretation of ORs, the probability of each treatment being the most effective or safest was calculated using a Bayesian approach, and probability values were estimated by the surface under the cumulative ranking (SUCRA) curve and the rank of each intervention [46, 47]. Cluster analyses were used to group treatments according to their similarity with regard to both outcomes [46]. All analyses were executed using R (V.3.2.1) package gemtc (V.0.6) with the Markov Chain Monte Carlo engine Open BUGS (V.3.4.0).

## SUPPLEMENTARY MATERIALS FIGURES AND TABLES




