# Supplementary material for: A network meta-analysis of eight chemotherapy regimens for treatment of advanced ovarian cancer
Source: Oncotarget. 2016 Nov 9;8(12):19125–36. doi: 10.18632/oncotarget.13253 (PMC5386673; doi:10.18632/oncotarget.13253)
Supplement: Supplementary file 1 [file oncotarget-08-19125-s001.pdf]

A network meta-analysis of eight chemotherapy regimens for treatment of advanced ovarian cancer

SUPPLEMENTARY FIGURES AND TABLES

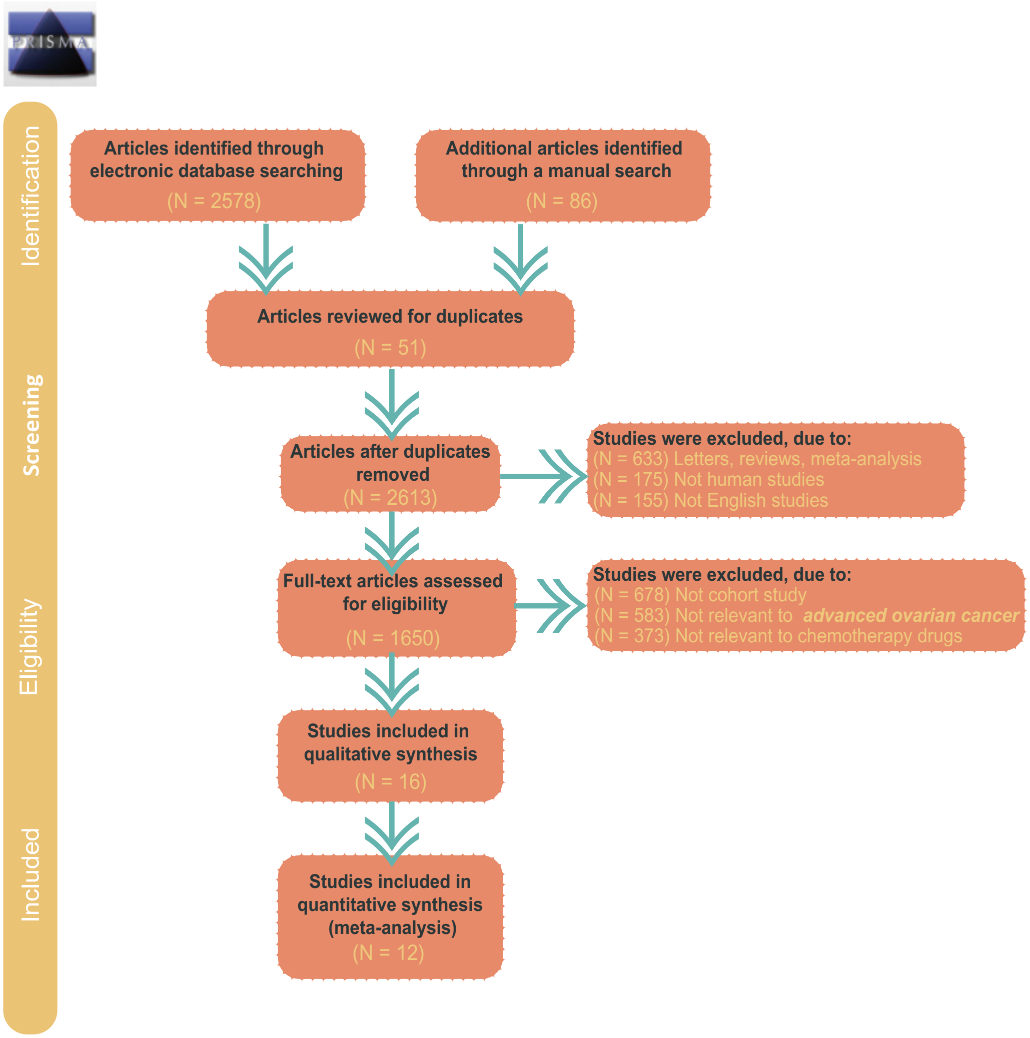

Supplementary Figure 1: Flow diagram showing the reference screening process.

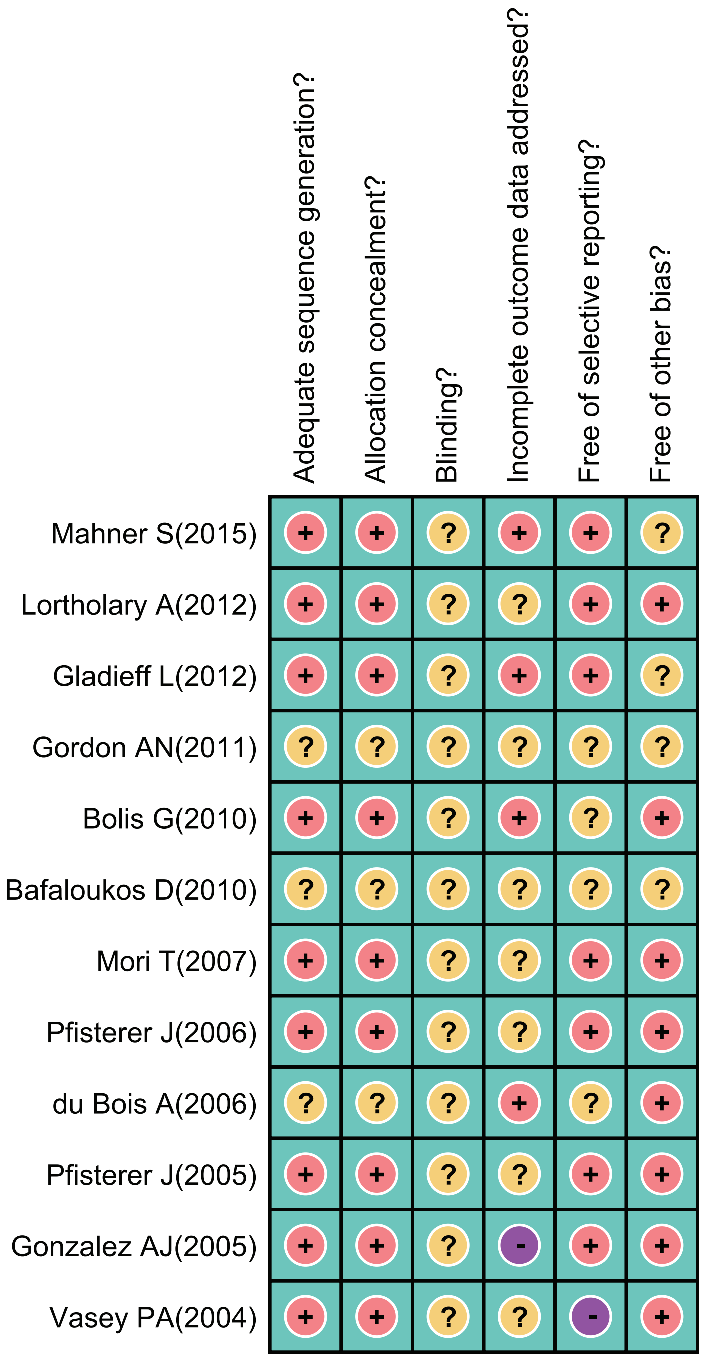

Supplementary Figure 2: Assessment of bias using the Cochrane Collaboration's tool.

Supplementary Table 1: The baseline characteristics for included studies

See Supplementary File 1

Supplementary Table 2: Estimated OR and 95%CI from pairwise meta-analysis of efficacy events in advanced ovarian cancer patients in terms of CR, PR and SD

See Supplementary File 1

Supplementary Table 3: Odds ratios and 95% confidence intervals of eight drugs in the treatment of advanced ovarian cancer in terms of CR, PR and SD

See Supplementary File 1
