# Supplementary material for: A network meta-analysis of eight chemotherapy regimens for treatment of advanced ovarian cancer
Source: Oncotarget. 2016 Nov 9;8(12):19125–36. doi: 10.18632/oncotarget.13253 (PMC5386673; doi:10.18632/oncotarget.13253)
Supplement: Supplementary file 2 [file oncotarget-08-19125-s002.docx]

**Supplement Table S1. The baseline characteristics for included studies.**

| **First author** | **Year** | **Country** | **Clinical trial stages** | **Interventions** | | **N** | **n** | |  |  | | **Patients age (years)** | |  | |
| --- | --- | --- | --- | --- | --- | --- | --- | --- | --- | --- | --- | --- | --- | --- | --- |
|  |  |  |  | **T1** | **T2** |  | **T1** | **T2** | | | **T1** | | **T2** | |  |
| Mahner S | 2015 | Germany | Phase III | A | D | 259 | 128 | 131 | | | 63(27-82) | | 60(30-80) | |  |
| Lortholary A | 2012 | France | Phase II | A | E | 108 | 51 | 57 | | | 60 (43–77) | | 60 (30–80) | |  |
| Gladieff L | 2012 | France | Phase III | A | D | 344 | 183 | 161 | | | 60 (30–80) | | 60 (24–82) | |  |
| Gordon AN | 2011 | USA | Phase III | A | B | 831 | 414 | 417 | | | 60 (22–86) | | 60 (22–84) | |  |
| Bolis G | 2010 | Italy | Phase III | A | F | 326 | 170 | 156 | | | 57.4 ± 10.2 | | 58.7 ± 9.4 | |  |
| Bafaloukos D | 2010 | Greece | Phase II | A | D | 189 | 96 | 93 | | | 63 (37-81) | | 62 (38-89) | |  |
| Mori T | 2007 | Japan | Phase II | A | H | 29 | 16 | 13 | | | 54.9 | | 57.7 | |  |
| Pfisterer J | 2006 | Germany | Phase III | A | F | 1308 | 650 | 658 | | | 60 (20 – 81) | | 60 (20 – 81) | |  |
| du Bois A | 2006 | Germany | Phase III | A | G | 1282 | 635 | 647 | | | 58(22-79) | | 60(21-79) | |  |
| Pfisterer J | 2005 | Germany | Phase III | B | C | 356 | 178 | 178 | | | 58.1 (36–78) | | 56.5 (21–81) | |  |
| Gonzalez-Martin AJ | 2005 | Spain | Phase II | A | C | 78 | 38 | 40 | | | 59 (40–77) | | 61 (35–77) | |  |
| Vasey PA | 2004 | UK | Phase III | A | H | 1077 | 538 | 539 | | | 59 (19–84) | | 59 (21–85) | |  |

T=treatment; A= Paclitaxel+Carboplatin; B= Gemcitabine+Carboplatin; C= Carboplatin; D= Pegylated liposomal doxorubicin+Carboplatin; E= Paclitaxel; F= Paclitaxel+Carboplatin+Topotecan; G= Paclitaxel+ Carboplatin +Epirubicin; H= Docetaxel+Carboplatin

**Supplement Table S2. Estimated OR and 95%CI from pairwise meta-analysis of efficacy events in advanced ovarian cancer patients in terms of CR, PR and SD.**

| **Included studies** | **Comparisons** | **Efficacy events** | |  | **Pairwise meta-analysis** | | |
| --- | --- | --- | --- | --- | --- | --- | --- |
|  |  | **Treatment1** | **Treatment2** |  | **OR (95%CI)** | ***I*^2^** | ***P_h_*** |
| **CR** | | | | | | | |
| Gordon AN(2011)^[23]^ | A vs. B | 50/114 | 57/139 |  | 1.12 (0.68-1.86) | NA | NA |
| Gonzalez-Martin AJ(2005) ^[30]^ | A vs. C | 11/41 | 8/40 |  | 1.17 (0.52-4.14) | NA | NA |
| Gladieff L(2012)^[22]^ | A vs. D | 47/279 | 41/254 |  | 1.07 (0.38-3.03) | 78.4% | 0.031 |
| Bafaloukos D(2010)^[25]^ | A vs. D |  |  |  |  |  |  |
| Lortholary A(2012)^[21]^ | A vs. E | 7/54 | 3/57 |  | 2.68 (0.66-10.96) | NA | NA |
| Bolis G(2010)^[24]^ | A vs. F | 129/820 | 122/814 |  | 1.02 (0.77-1.36) | 0% | 0.4091 |
| Pfisterer J(2006) ^[27]^ | A vs. F |  |  |  |  |  |  |
| du Bois A(2006) ^[28]^ | A vs. G | 70/635 | 57/647 |  | 1.28 (0.89-1.85) | NA | NA |
| Mori T(2007) ^[26]^ | A vs. H | 86/312 | 86/313 |  | 1.01 (0.71-1.44) | 0.0% | 0.816 |
| Vasey PA(2004) ^[31]^ | A vs. H |  |  |  |  |  |  |
| Pfisterer J(2005) ^[29]^ | B vs. C | 26/178 | 11/178 |  | **2.60 (1.24-5.43)** | NA | NA |
| **PR** | | | | | | | |
| Gordon AN(2011)^[23]^ | A vs. B | 31/114 | 37/139 |  | 1.03 (0.59-1.80) | NA | NA |
| Gonzalez-Martin AJ(2005) ^[30]^ | A vs. C | 20/41 | 12/40 |  | 2.22 (0.89-5.53) | NA | NA |
| Gladieff L(2012)^[22]^ | A vs. D | 91/279 | 70/254 |  | 1.19 (0.61-2.32) | 64.7% | 0.092 |
| Bafaloukos D(2010)^[25]^ | A vs. D |  |  |  |  |  |  |
| Lortholary A(2012)^[21]^ | A vs. E | 12/24 | 17/30 |  | 0.76 (0.26-2.25) | NA | NA |
| Bolis G(2010)^[24]^ | A vs. F | 93/820 | 87/814 |  | 1.04 (0.75-1.43) | 0% | 0.418 |
| Pfisterer J(2006) ^[27]^ | A vs. F |  |  |  |  |  |  |
| du Bois A(2006) ^[28]^ | A vs. G | 41/635 | 44/647 |  | 0.95 (0.61-1.47) | NA | NA |
| Mori T(2007) ^[26]^ | A vs. H | 94/312 | 93/313 |  | 1.03 (0.73-1.45) | 0.0% | 0.436 |
| Vasey PA(2004) ^[31]^ | A vs. H |  |  |  |  |  |  |
| Pfisterer J(2005) ^[29]^ | B vs. C | 58/178 | 44/178 |  | 1.47 (0.93-2.34) | NA | NA |
| **SD** | | | | | | | |
| Gordon AN(2011)^[23]^ | A vs. B | 16/114 | 22/139 |  | 0.87 (0.43-1.74) | NA | NA |
| Gonzalez-Martin AJ(2005) ^[30]^ | A vs. C | 2/41 | 5/40 |  | 0.36 (0.07-1.97) | NA | NA |
| Gladieff L(2012)^[22]^ | A vs. D | 77/279 | 79/254 |  | 0.82 (0.56-1.20) | 0% | 0.4845 |
| Bafaloukos D(2010)^[25]^ | A vs. D |  |  |  |  |  |  |
| Lortholary A(2012)^[21]^ | A vs. E | 15/51 | 13/57 |  | 1.41 (0.59-3.34) | NA | NA |
| Bolis G(2010)^[24]^ | A vs. F | 34/820 | 31/814 |  | 1.29 (0.37-4.52) | 79.2% | 0.028 |
| Pfisterer J(2006) ^[27]^ | A vs. F |  |  |  |  |  |  |
| du Bois A(2006) ^[28]^ | A vs. G | 28/635 | 15/647 |  | **1.94 (1.03-3.67)** | NA | NA |
| Mori T(2007) ^[26]^ | A vs. H | 2/16 | 1/13 |  | 1.71 (0.14-21.33) | NA | NA |
| Pfisterer J(2005) ^[29]^ | B vs. C | 68/178 | 69/178 |  | 0.98 (0.64-1.50) | NA | NA |

Notes: CR=complete response; PR=partial response; SD=stable disease; OR=odd ratios; 95%CI=95% confidence intervals; NA=not available; T=treatment; A= Paclitaxel+Carboplatin; B= Gemcitabine+Carboplatin; C= Carboplatin; D= Pegylated liposomal doxorubicin+Carboplatin; E= Paclitaxel; F= Paclitaxel+Carboplatin+Topotecan; G= Paclitaxel+ Carboplatin +Epirubicin; H= Docetaxel+Carboplatin

.

**Supplement Table S3. Odds ratios and 95% confidence intervals of eight drugs in the treatment of advanced ovarian cancer in terms of CR, PR and SD.**

| **Odds ratios (95% confidence intervals)** | | | | | | | |
| --- | --- | --- | --- | --- | --- | --- | --- |
| **CR** | | | | | | | |
| **PC** | 0.80 (0.38, 1.65) | 0.48 (0.17, 1.55) | 0.92 (0.40, 2.18) | 0.35 (0.05, 1.84) | 0.99 (0.46, 2.18) | 0.78 (0.26, 2.28) | 0.98 (0.37, 2.52) |
| 0.98 (0.36, 2.44) | **GC** | 0.47 (0.16, 1.35) | 0.90 (0.24, 3.11) | 0.34 (0.04, 2.29) | 0.96 (0.27, 3.16) | 0.77 (0.18, 3.03) | 0.96 (0.24, 3.46) |
| 2.06 (0.65, 6.05) | 2.12 (0.74, 6.25) | **Carboplatin** | 1.92 (0.45, 7.14) | 0.73 (0.08, 5.23) | 2.05 (0.50, 7.58) | 1.62 (0.32, 7.06) | 2.01 (0.44, 8.17) |
| 1.09 (0.46, 2.49) | 1.11 (0.32, 4.16) | 0.52 (0.14, 2.24) | **PLD+ Carboplatin** | 0.38 (0.05, 2.54) | 1.07 (0.34, 3.37) | 0.85 (0.21, 3.33) | 1.06 (0.29, 3.68) |
| 2.85 (0.54, 18.26) | 2.95 (0.44, 24.60) | 1.37 (0.19, 13.07) | 2.63 (0.39, 20.63) | **Paclitaxel** | 2.86 (0.45, 21.22) | 2.25 (0.30, 18.39) | 2.80 (0.41, 21.78) |
| 1.02 (0.46, 2.20) | 1.04 (0.32, 3.74) | 0.49 (0.13, 2.02) | 0.93 (0.30, 2.95) | 0.35 (0.05, 2.22) | **PC+Topotecan** | 0.79 (0.21, 2.94) | 1.00 (0.29, 3.20) |
| 1.28 (0.44, 3.86) | 1.31 (0.33, 5.63) | 0.62 (0.14, 3.14) | 1.18 (0.30, 4.83) | 0.44 (0.05, 3.32) | 1.26 (0.34, 4.82) | **PC+Epirubicin** | 1.24 (0.30, 5.11) |
| 1.02 (0.40, 2.67) | 1.05 (0.29, 4.10) | 0.50 (0.12, 2.26) | 0.94 (0.27, 3.40) | 0.36 (0.05, 2.47) | 1.00 (0.31, 3.42) | 0.80 (0.20, 3.30) | **DC** |
| **PR** | | | | | | | |
| **PC** | 0.90 (0.39, 1.88) | 0.56 (0.22, 1.26) | 0.82 (0.44, 1.62) | 1.29 (0.32, 4.98) | 0.97 (0.53, 1.83) | 1.07 (0.45, 2.55) | 1.03 (0.52, 2.41) |
| 1.12 (0.53, 2.54) | **GC** | 0.62 (0.29, 1.30) | 0.92 (0.35, 2.69) | 1.45 (0.31, 7.25) | 1.09 (0.42, 3.19) | 1.21 (0.36, 4.00) | 1.18 (0.44, 4.02) |
| 1.80 (0.80, 4.49) | 1.61 (0.77, 3.47) | **Carboplatin** | 1.48 (0.54, 4.67) | 2.33 (0.50, 12.50) | 1.75 (0.67, 5.37) | 1.96 (0.58, 6.73) | 1.86 (0.66, 6.75) |
| 1.22 (0.62, 2.26) | 1.09 (0.37, 2.87) | 0.68 (0.21, 1.85) | **PLD+ Carboplatin** | 1.56 (0.31, 6.75) | 1.20 (0.49, 2.91) | 1.34 (0.41, 3.80) | 1.27 (0.50, 3.59) |
| 0.78 (0.20, 3.14) | 0.69 (0.14, 3.22) | 0.43 (0.08, 2.01) | 0.64 (0.15, 3.18) | **Paclitaxel** | 0.75 (0.18, 3.34) | 0.85 (0.17, 3.92) | 0.80 (0.18, 3.81) |
| 1.03 (0.55, 1.87) | 0.92 (0.31, 2.35) | 0.57 (0.19, 1.50) | 0.84 (0.34, 2.05) | 1.33 (0.30, 5.63) | **PC+Topotecan** | 1.11 (0.37, 3.19) | 1.07 (0.42, 2.98) |
| 0.93 (0.39, 2.25) | 0.83 (0.25, 2.74) | 0.51 (0.15, 1.71) | 0.74 (0.26, 2.43) | 1.18 (0.26, 6.02) | 0.90 (0.31, 2.72) | **PC+Epirubicin** | 0.96 (0.33, 3.33) |
| 0.97 (0.42, 1.93) | 0.85 (0.25, 2.29) | 0.54 (0.15, 1.51) | 0.79 (0.28, 2.01) | 1.25 (0.26, 5.45) | 0.94 (0.34, 2.37) | 1.04 (0.30, 3.07) | **DC** |
| **SD** | | | | | | | |
| **PC** | 1.41 (0.45, 4.42) | 1.65 (0.47, 6.68) | 1.17 (0.46, 2.80) | 0.73 (0.18, 2.83) | 0.84 (0.30, 2.06) | 0.51 (0.14, 1.90) | 0.52 (0.02, 8.14) |
| 0.71 (0.23, 2.22) | **GC** | 1.15 (0.43, 3.56) | 0.82 (0.19, 3.40) | 0.50 (0.09, 3.02) | 0.58 (0.13, 2.45) | 0.36 (0.07, 2.01) | 0.37 (0.01, 6.87) |
| 0.61 (0.15, 2.14) | 0.87 (0.28, 2.34) | **Carboplatin** | 0.70 (0.14, 3.15) | 0.43 (0.06, 2.86) | 0.50 (0.09, 2.31) | 0.31 (0.05, 1.81) | 0.31 (0.01, 5.96) |
| 0.86 (0.36, 2.16) | 1.22 (0.29, 5.31) | 1.42 (0.32, 7.23) | **PLD+ Carboplatin** | 0.63 (0.12, 3.20) | 0.72 (0.18, 2.53) | 0.44 (0.09, 2.23) | 0.45 (0.01, 7.78) |
| 1.37 (0.35, 5.59) | 1.99 (0.33, 11.74) | 2.35 (0.35, 16.82) | 1.59 (0.31, 8.35) | **Paclitaxel** | 1.15 (0.21, 5.88) | 0.70 (0.11, 5.04) | 0.71 (0.02, 18.19) |
| 1.18 (0.48, 3.31) | 1.72 (0.41, 7.91) | 2.00 (0.43, 11.13) | 1.38 (0.40, 5.47) | 0.87 (0.17, 4.85) | **PC+Topotecan** | 0.61 (0.13, 3.24) | 0.62 (0.02, 11.52) |
| 1.96 (0.53, 7.03) | 2.80 (0.50, 14.98) | 3.26 (0.55, 21.34) | 2.29 (0.45, 10.87) | 1.43 (0.20, 8.91) | 1.63 (0.31, 7.77) | **PC+Epirubicin** | 0.98 (0.03, 19.34) |
| 1.92 (0.12, 56.78) | 2.74 (0.15, 94.34) | 3.20 (0.17, 120.62) | 2.23 (0.13, 67.05) | 1.41 (0.05, 48.45) | 1.62 (0.09, 51.18) | 1.02 (0.05, 33.42) | **DC** |

**Notes:** Odds ratios and 95% confidence intervals below the treatments should be read from row to column while above the treatments should be read from column to row. CR=complete response; PR=partial response; SD=stable disease; PC= Paclitaxel+Carboplatin; GC= Gemcitabine+Carboplatin; PLD= Pegylated liposomal doxorubicin; DC= Docetaxel+Carboplatin
